# Dopaminergic Neuron‐Derived AIMP1 Promotes Neurodegeneration via CD23‐Dependent Microglial Activation

**DOI:** 10.1111/cns.70472

**Published:** 2025-06-16

**Authors:** Qinqin Wang, Hao Yu, Xunan Yuan, Ruolin Li, Xuezhi Li, Shu Yin, Xiaodan Ma, Xinmiao Wang

**Affiliations:** ^1^ Institute of Mental Health Jining Medical University Jining China; ^2^ Affiliated Hospital of Jining Medical University Jining Shandong China; ^3^ School of Health Management Xihua University Chengdu China

**Keywords:** AIMP1, DA neuron, microglia, neuroinflammation, Parkinson's disease

## Abstract

**Background:**

Parkinson's disease (PD), the second most prevalent age‐associated neurodegenerative disorder, is characterized by the degeneration and loss of dopaminergic (DA) neurons in the substantia nigra pars compacta (SN). Among the intricate pathophysiological processes of PD, chronic neuroinflammation has emerged as a pivotal hallmark in the pathogenesis of PD. The aminoacyl tRNA synthetase complex has been reported to play an important role in modulating the immune response and associated diseases. Nevertheless, the specific functions and implications of the complex in PD remain largely unclear.

**Methods:**

Enzyme‐linked immunosorbent assay (ELISA) was used to investigate levels of AIMP1 and TNF‐α. An in vivo PD model was constructed by administering 1‐Methyl‐4‐phenyl‐1,2,3,6‐tetrahydropyridine (MPTP) in mice. A PD cell model was established by treating SH‐SY5Y cells with 1‐methyl‐4‐phenylpyridinium (MPP^+^). Pole test was performed to assess the motor function of mice. DA neuron survival and microglia activation were detected by immunofluorescence. Western blot and qPCR were used to detect the levels of tyrosine hydroxylase (TH) and inflammatory cytokines. RNA‐Seq analysis was performed to explore possible mechanisms.

**Results:**

The levels of AIMP1, a co‐factor of the aminoacyl tRNA synthetase complex, were significantly elevated in the blood of PD patients. *Aimp1* knockout or knockdown remarkably improved the viability of DA neurons in the MPTP‐induced mouse model of PD. *Aimp1* deficiency reduced microglial activation in PD mice. RNA‐Seq analysis revealed that AIMP1 promoted microglial inflammatory response. Moreover, the AIMP1‐induced microglial activation was CD23 dependent.

**Conclusions:**

Collectively, our findings indicate that AIMP1 derived from DA neurons exacerbates neuroinflammation, promotes the death of DA neurons and contributes to the development of PD. This study offers novel insights into the molecular mechanisms underlying PD and blocking the AIMP1‐CD23 signaling pathway potentially serves as a therapeutic strategy for PD.

## Graphical abstract

AIMP1‐derived from DA neurons augmented microglia‐associated neuroinflammation, which was CD23‐dependent, promoting the death of DA neurons and contributing to the development of PD.

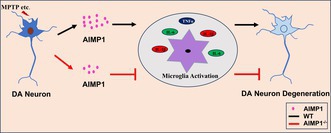

## 1 Introduction

Parkinson's disease (PD) is an age‐related neurodegenerative disease [1]. Degeneration of DA neurons in the SN was a crucial pathological hallmark of PD [1, 2]. Extensive investigations have focused on elucidating the molecular mechanisms underlying this selective DA neuronal loss. Multiple factors, such as gene mutation, autophagy dysfunctions, and mitochondria impairments, have been implicated in the pathogenesis of PD [3, 4]. Emerging research highlights neuroinflammation as an important driver of PD progression [5, 6]. Microglia have been recognized as the principal initiator of neuroinflammation, underscoring their essential role in the development and progression of this process [7, 8]. An increasing number of studies have proposed that mitigating the pro‐inflammatory response mediated by microglia significantly protects DA neurons from death in PD [9, 10].

AIMP1 functioned as an auxiliary factor within the huge macromolecular aminoacyl tRNA synthetase complex [11]. Classically, AIMP1 was indicated as a scaffolding protein, contributing to the translation process [11]. A growing body of studies has elucidated that AIMP1 exerted regulatory effects on various physiological processes such as angiogenesis and immune response [12]. Studies have also shown that AIMP1 participated in pathological progression such as Alzheimer ‘s disease (AD) and cancer [13, 14]. Nevertheless, the potential functions and implications of AIMP1 in PD remained largely unknown.

In this study, we observed a marked elevation of AIMP1 levels both in the blood samples of PD patients and the supernatant of a PD cell model. Genetic ablation of *Aimp1* conferred substantial neuroprotection against dopaminergic neuronal loss in PD mouse models. Additionally, AIMP1 knockdown led to a substantial improvement in the motor ability of the MPTP‐induced PD mice model. Intriguingly, AIMP1 deficiency resulted in decreased microglia activation in PD mice, indicating the potential regulatory roles of AIMP1 in microglial‐associated neuroinflammation. Furthermore, through RNA sequencing, it was revealed that AIMP1 promoted neuroinflammation by upregulating pro‐inflammatory cytokines such as interleukin‐1β (IL‐1β) and simultaneously downregulating the anti‐inflammatory factors like arginase1 (Arg1). Moreover, AIMP1 mediated the microglial inflammatory response via CD23. Notably, conditional knockout of AIMP1 specifically in microglia or astrocytes failed to elicit significant alterations in the number of DA neurons in a mouse model of PD. Our findings established neuron‐derived AIMP1 as a novel regulator of microglial‐dependent neuroinflammation in PD pathogenesis. This study broadened the current understanding of the function of AIMP1 and the pathological mechanisms of PD, providing novel perspectives for the development of new therapeutic strategies for PD.

## 2 Materials and Methods

### 2.1 Human Participants

The study was approved by the Research Ethics Committee of Jining.

Medical University (JNMC‐2023‐YX‐014). Informed consent was obtained from all participants, including 4 healthy controls and 5 Parkinson's disease patients recruited from the Affiliated Hospital of Jining Medical University. The investigation strictly adhered to ethical guidelines. All the clinicopathological information of these participants was comprehensively presented in Table S1.

### 2.2 Measurement of Blood Levels of AIMP1

Peripheral blood samples were collected from healthy controls and PD patients. These samples were subsequently analyzed using an enzyme‐linked immunosorbent assay (Elisa) kit (Cloud‐Clone Corp.). In brief, the human whole blood from the participants was dispensed into the 96‐well plate provided in the kit. Following 1 h incubation, the plate was washed according to the protocol. The absorbance values were then measured at a wavelength of 450 nm.

### 2.3 Mice

*Aimp1* knockout (AIMP1 knockout, AIMP1 KO, *AIMP1* ^−/−^), hGFAP‐CreER^T2^, CD11B‐Cre, and AIMP1‐floxed transgenic mice were kindly provided by Prof. Jiawei Zhou (CAS Center for Excellence in Brain Science and Intelligence Technology, Shanghai). C57BL/6 mice were purchased from Jinan Pengyue laboratory animal breeding Co.Ltd. These mice were housed under a 12‐h light/dark cycle at a temperature of 23°C with food and water available *ad libitum*. All experimental procedures complied with the Animal Welfare Act and the NIH Guide for the Care and Use of Laboratory Animals.

### 2.4 Animal Model

The AIMP1 KO and conditional KO adult mice were injected with MPTP at a dose of 20 mg/kg (for female mice, the dose was 15 mg/kg), with four injections administered within 8 h. These mice were sacrificed 1 week after MPTP injection. C57BL/6 wild‐type adult mice were administered MPTP (30 mg/kg) for 5 consecutive days after lentivirus challenge. Behavioral tests were conducted 1 week after the last MPTP injection, and the mice were sacrificed 10–14 days after the final injection.

### 2.5 Primary Astrocytes and Microglia Culture

We cultured the astrocytes and microglia according to the protocol described previously [15]. In brief, the cortex of neonatal mice, age 1–3 days, was dissociated and trypsinized. Cells were plated on the poly‐L‐lysine (PLL) pre‐treated flasks. The cell mixture was shaken for 1 h at 37°C with 250 rpm to get microglia in the supernatant, followed by shaking at 250 rpm for 16 h to remove NG2 glial cells in the supernatant. The remaining cells were collected for astrocyte culture. The isolated microglia and astrocytes were cultured in DMEM/F12 medium containing 10% fetal bovine serum.

### 2.6 BV2 Cell Culture and CM Collection

BV2 cell was cultured in DMEM supplemented with 10% fetal bovine serum, maintained in a 5% CO_2_ atmosphere at 37°C. These cells were then passaged into 12‐well plates. When the cells reached 50%–60% confluency, they were treated with AIMP1 at a final concentration of 0.5 μg/mL. Following a 24‐h incubation with AIMP1, cells were collected for western blot analysis and RNA quantification. The culture medium of AIMP1‐treated BV2 cells was centrifuged at 3000 g for 5 min and the supernatant was collected as conditioned medium (CM).

### 2.7 RNA‐Seq Analysis

The protocol for analysis of RNA‐Seq was carried out by Shanghai Applied Protein Technology. In brief, RNA was isolated from control and AIMP1‐treated BV2 cells which had been challenged by AIMP1 for 24 h. RNA samples were assessed using the Nanodrop ND‐2000 (Thermo Scientific, USA). Qualified samples were used for the library construction. ABclonal mRNA‐seq Lib Prep Kit (ABclonal, China) was used to prepare the library and the quality of the library was evaluated by the Agilent Bioanalyzer 4150 system. Sequencing was conducted on an Illumina Novaseq 6000/MGISEQ‐T7 instrument. The data was produced from the platform of Illumina/BGI and bioinformatics analysis was performed based on the generated data.

### 2.8 Peptides, Regents and Antibodies

AIMP1 was purchased from MCE (HY‐P77643). MPTP was obtained from MedChem Express (HY‐15608). Anti‐AIMP1 antibody was a gift from Prof. Jiawei Zhou (CAS Center for Excellence in Brain Science and Intelligence Technology, Shanghai). ElisaKits were purchased from Cloud‐Clone Corp. (SEL761Mu) and Bioswamp (HM11704). MPP^+^ was purchased from Glpbio (GC18188). Primary antibodies included: Anti‐β‐actin antibody (ZSGB‐BIO, TA‐09); Anti‐TH antibody (Millipore, MAB318); Anti‐AIMP1 antibody (Cloud‐Clone Corp, PAL761Mu01); Anti‐tumor necrosis factor (TNF)‐α antibody (Proteintech, 17590‐1‐AP). The corresponding secondary antibodies used were as follows: HRP‐conjugated goat anti‐mouse IgG (ZSGB‐BIO, ZB‐2305); HRP‐conjugated goat anti‐rabbit IgG (ZSGB‐BIO, ZB‐2301).

### 2.9 Stereotaxic Injection and AIMP1 Knockdown

To achieve AIMP1 knockdown in wild type mice, a lentiviral vector containing the shRNA sequence was constructed (OBiO, Shanghai, China). The mice under anesthesia received the stereotaxic injection of the lentiviral vector in the SN with the coordinates (AP: −4.4 mm, ML:1.2 mm, DV: 4.8 mm from the bregma) with a Hamilton 5 μL syringe at the rate of 0.4 μL/min. The syringe was gradually withdrawn 5–10 min after the completion of the injection.

### 2.10 Generation of CD23‐Knockdown BV2 Microglia

To knock down CD23 in BV2 microglial cells, lentiviral vectors encoding shRNA targeting CD23 (shRNA‐CD23) were constructed (OBiO Technology, Shanghai, China). Cells were transduced with shRNA‐CD23 by lentiviral transfection. Stably transduced BV2 cells were subsequently treated with AIMP1 following antibiotic selection. Cells underwent harvest 24 h post‐treatment for quantification of inflammatory cytokine expression.

### 2.11 Western Blot and Quantification

Western blot analysis was performed as previously described [16]. Briefly, protein lysates from brain tissue and cell lines were separated by SDS‐PAGE gels. Primary antibodies employed were: β‐actin (1:5000), TH (1:1000), IL‐1β (1:1000) and TNFα (1:1000). After overnight incubation with primary antibodies, membranes were washed three times with 1 × TBST and incubated with HRP‐conjugated secondary antibodies (goat anti‐rabbit IgG (1:10,000) and goat anti‐mouse IgG (1:10,000)) at room temperature for 1–2 h. Chemiluminescent signals were detected using ECL substrate (Multi Sciences, P1425). Image J was used to quantify the protein levels by measuring band optical density, with β‐actin serving as the control.

### 2.12 RNA Isolation and Quantitative RT‐PCR

Total RNA was extracted following the previously described protocol [16]. Briefly, RNA was extracted from brain tissue and cultured cells using Trizol reagent. 1 μg RNA was used to synthesize cDNA with the reverse transcription Kit (Best Enzymes Biotech Co. Ltd., EG15133S) in accordance with the manufacturer's instructions. Quantitative Real‐time polymerase chain reaction (Quantitative RT‐PCR) was carried out using the Kit (Best Enzymes Biotech Co. Ltd., EG20117M) on the QuantStudio 5 Real‐Time PCR System (QuantStudio5, applied biosystems by Thermo Fisher Scientific). The primers for qPCR were as follows: β‐actin: Forward: 5′‐CGTCGACAACGGCTCCGGCATG‐3′, Reverse: 5′‐CCACCATCACACCCTGGTGCCTAGG‐3′; IL‐1β, Forward: 5′‐AGGAGAACCAAGCAACGACA‐3′, Reverse: 5′‐CTTGGGATCCACACTCTCCAG‐3′; IL6, Forward: 5′‐GCCTTCTTGGGACTGATGCT‐3′, Reverse:5′‐TGCCATTGCACAACTCTTTTCT‐3′; IL‐12β, Forward: 5′‐TGGTTTGCCATCGTTTTGCTG‐3′, Reverse:5′‐ACAGGTGAGGTTCACTGTTTCT‐3′; TNFα, Forward: 5′‐ACGTCGTAGCAAACCACCAA‐3′, Reverse:5′‐ATAGCAAATCGGCTGACGGT‐3′;

Arg1, Forward: 5′‐AGCTCTGGGAATCTGCATGG‐3′, Reverse:5′‐ATCGGCCTTTTCTTCCTTCCC‐3′; CD206, Forward: 5′‐GATGACCTGTGCTCGAGAGG‐3′, Reverse:5′‐TCTCGCTTCCCTCAAAGTGC‐3′; Csf1r, Forward: 5′‐GGTTGTAGAGCCGGGTGAAA‐3′, Reverse:5′‐AAGAGTGGGCCGGATCTTTG‐3′;

Cxcl2, Forward: 5′‐CCCAGACAGAAGTCATAGCCAC‐3′, Reverse:5′‐CGAGGCACATCAGGTACGAT‐3′; CX3CR1, Forward: 5′‐CCATCTGCTCAGGACCTCAC‐3′, Reverse:5′‐CACCAGACCGAACGTGAAGA‐3′;

Ccl2, Forward: 5′‐AGCCAACTCTCACTGAAGCC‐3′, Reverse:5′‐AGCTTGGTGACAAAAACTACAGC‐3′; Ptgs2, Forward: 5′‐CCCATGGGTGTGAAGGGAAAT‐3′, Reverse:5′‐TCCATCCTTGAAAAGGCGCA‐3′.

### 2.13 Immunofluorescence and Confocal Microscopy

Brian sections were immunostained with primary antibody, then washed the sections with 1 × PBST, followed by incubation with secondary antibody conjugated with Alexa488 or Alexa555. Fluorescence images were acquired using either a cooled CCD (DP72, Olympus) on a microscope (BX51; Olympus).

### 2.14 Tamoxifen Injection

Tamoxifen (TAM, Sigma‐Aldrich) was dissolved in a 95% corn oil (Sigma‐Aldrich)/5% ethanol solution with a concentration of 20 mg/mL. Adult mice received daily intraperitoneal injections of 80–100 μg TAM/kg body weight for 7 days. PD models were established at least 14 days post‐TAM administration.

### 2.15 Antibody Treatment

BV2 cells were maintained in DMEM supplemented with 10% fetal bovine serum under a humidified 5% CO_2_ atmosphere at 37°C. BV2 cells were seeded into 6/12‐well plates. At 50%–60% confluence, cells were stimulated with recombinant AIMP1 protein and anti‐CD23 antibody (CD23 Ab) at a concentration of 0.5 μg/mL. After 24 h treatment, cells were harvested for immunoblot analysis.

### 2.16 MTT Assay

SH‐SY5Y cells were passaged into a 96‐well plate. Upon reaching 60%–70% cell confluence, the cells were exposed to the CM from AIMP1‐treated BV2 cells. Following a 24‐h incubation, cell viability was assessed by the MTT assay performed according to the manufacturer's instructions of the kit (Bioswamp, BTK020). Finally, the absorbance at the wavelength of 570 nm was measured and recorded.

### 2.17 Pole Test

Pole test was performed 2 days after the final MPTP injection. Simply, a vertical wooden pole (approximately 40 cm in length) was positioned vertically in the mice cage. The animals were placed on the top of the pole and kept head downward. The research recorded the time of the animal to reach the pole bottom.

### 2.18 Elisa

BV2 cell was cultured in DMEM containing 10% fetal bovine serum under a humidified 5% CO_2_ atmosphere at 37°C. Cells were seeded into 6/12‐well plates. At 50%–60% confluence, cells were stimulated with AIMP1 and CD23 antibody (CD23 Ab) at the dose of 0.5 μg. After 24‐h treatment, culture supernatants were collected for Elisa. TNFα levels in supernatants were quantified according to the manufacturer's instructions (Bioswamp, MU30030).

### 2.19 Statistical Analysis

Statistical analysis was conducted with GraphPad software (GraphPad Prism; GraphPad Software). We assessed data normality using D'Agostino‐Pearson omnibus (K2)/Shapiro–Wilk (W)/Kolmogorov–Smirnov (distance) tests. The majority of datasets in our manuscript demonstrated normal/Gaussian distributions, and were submitted to *t* test or ANOVA followed by either Dunnet test, Student–Newman–Keul's test, or Sidak's multiple comparisons test (as a *post hoc* test). While specific datasets that deviated from normality were submitted to nonparametric analysis. Statistical significance was defined as *p* < 0.05. Data were expressed as mean ± SEM.

## 3 Results

### 3.1 Upregulation of AIMP1 in PD

**FIGURE 1:**
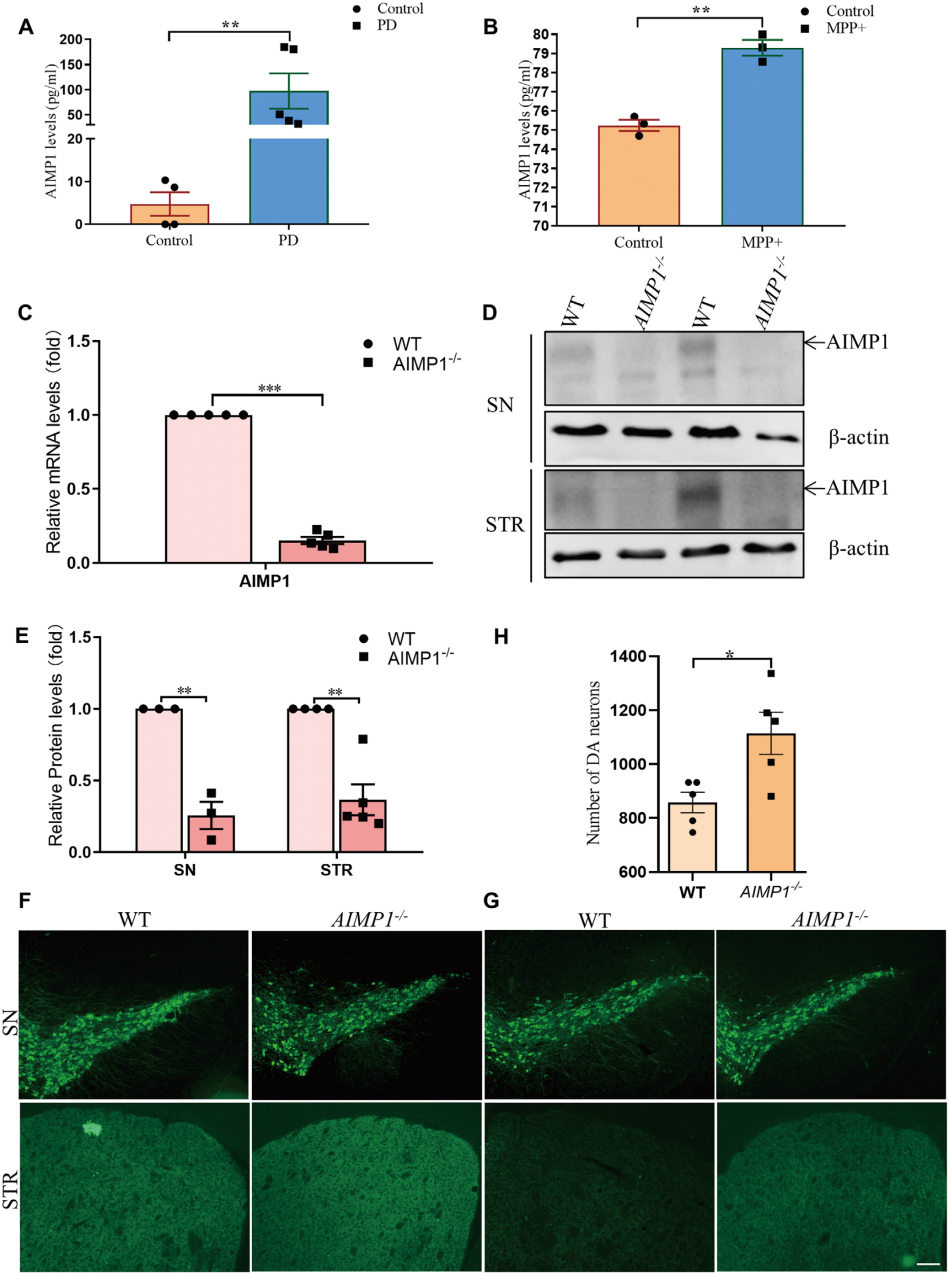
AIMP1 knockout protected DA neurons in PD model. (A) Increase of AIMP1 levels in the whole blood of PD patients. (B) Increase of AIMP1 levels in supernatant of SH‐SY5Y cell following MPP^+^ (500 μM; 24 h) treatment. (C) qPCR analysis of AIMP1 levels in adult WT and *AIMP1* ^*−/−*^ mice. (D) Representative blots of AIMP1 levels in adult WT and *AIMP1* ^*−/−*^ mice. (E) Quantification analysis of AIMP1 expression in the SN and STR shown in D. (F) Representative images of TH staining on SN and STR in adult WT and *AIMP1* ^*−/−*^ mice. (G) Representative images of TH staining on SN and STR in MPTP‐induced acute PD model of WT and *AIMP1* ^*−/−*^ mice (MPTP, 20 mg/kg for male mice, 15 mg/kg for female mice, i.p., 4 times in 1 day). (H) Quantification analysis of DA neuron (TH^+^) numbers in the SN of MPTP‐induced acute PD model shown in G. Unpaired *t* test. Data were expressed as mean ± SEM (*n* = 3–5). *p < 0.05, **p < 0.01, ***p < 0.001. Scale bar = 50 μm.

To investigate the correlation between AIMP1 and PD, we firstly quantified AIMP1 protein levels in the whole blood from PD patients and healthy controls. The demographical information of both healthy individuals and PD cases was summarized in Table S1. Elisa assay demonstrated a significant elevation in the blood levels of AIMP1 in PD patients compared to controls (Figure 1A). Subsequently, we established a PD cell model by treating the human neuroblastoma cell line, SH‐SY5Y, with 1‐methyl‐4‐phenylpyridinium (MPP^+^). Conditioned media analysis by Elisa revealed MPP^+^‐induced upregulation of AIMP1 protein levels in the supernatant of SH‐SY5Y cells (Figure 1B). These results indicated that the expression and secretion of AIMP1 derived from DA neurons were closely associated with PD‐related neurodegeneration.

### 3.2 AIMP1 Knockout Protected DA Neurons From Death in PD

To further investigate the role of AIMP1 in DA neuron neurodegeneration in a mouse model of PD, age‐matched adult wild‐type (WT) and AIMP1 knockout (*AIMP1* ^*−/−*^) mice were intraperitoneally administered MPTP. The knockout efficiency of AIMP1 was confirmed by qPCR and western blot, demonstrating that AIMP1 was efficiently knocked out at both transcriptional and translational levels (Figure 1C–E). Baseline immunohistochemical analysis indicated no significant alterations in DA neuron density in the SN or TH immunoreactivity in the STR between genotypes (Figure 1F). However, interestingly, AIMP1 deficiency significantly mitigated MPTP‐induced dopaminergic neurodegeneration (Figure 1G). Statistical analysis further corroborated the neuroprotective effects of AIMP1 deficiency in PD (Figure 1H).

### 3.3 AIMP1 Knockdown Improved the Behavioral and Pathological Performance of PD Mice

**FIGURE 2:**
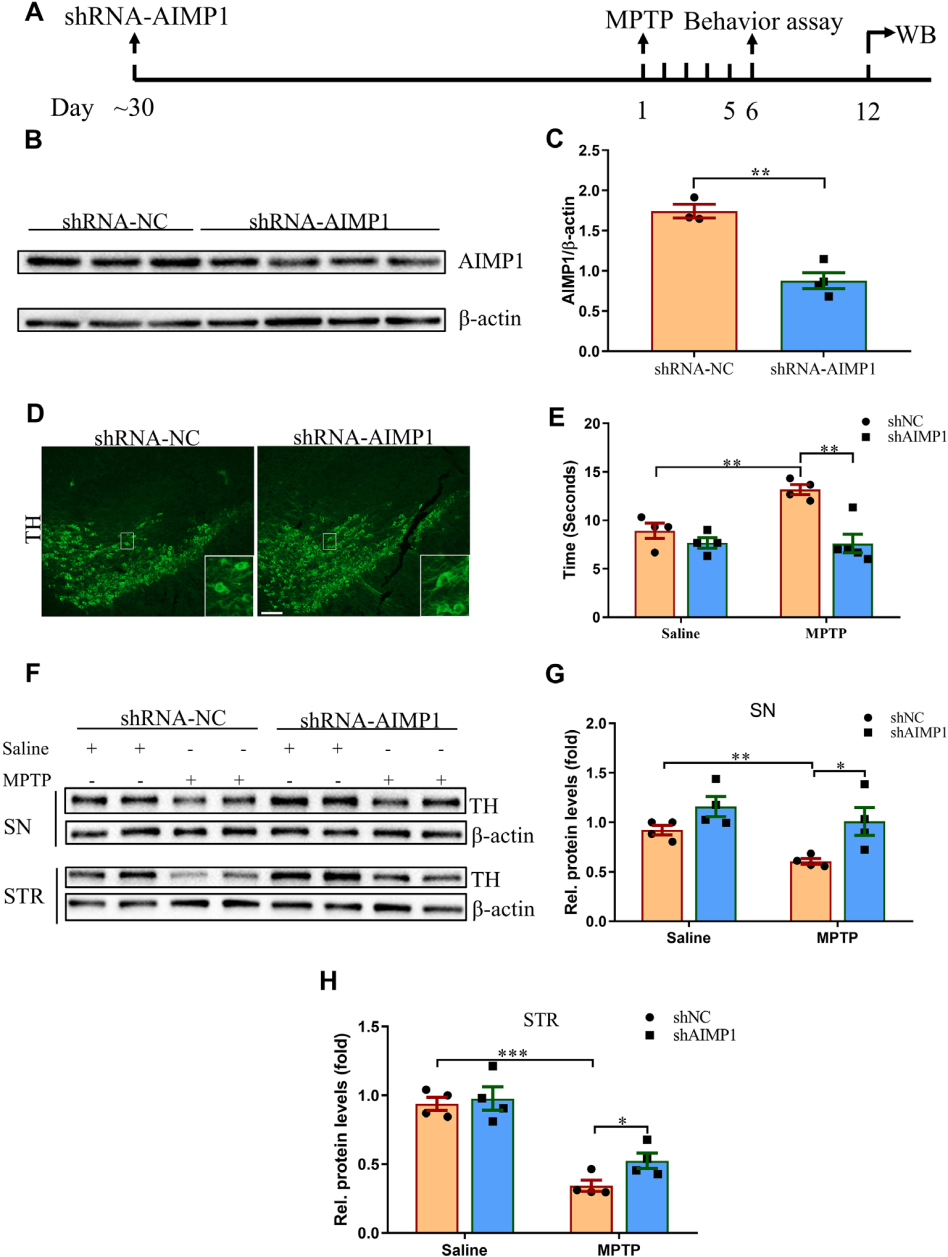
AIMP1 knockdown exerted neuroprotective roles in PD model. (A) Schematic diagram of AIMP1 knockdown experiments using shRNA in this study. (B) Representative immunoblots of AIMP1 levels in the SN after shAIMP1 stereotaxic injection. (C) Quantification analysis of AIMP1 expression levels in (B). (D) Representative images of DA neurons (TH^+^, green) in SN after shRNA‐AIMP1 (shAIMP1) or shRNA‐NC (shNC) stereotaxic injection. The enlarged view of the rectangular area of the corresponding image was shown in the bottom right corner. (E) Pole test data of PD mice after AIMP1 knockdown (MPTP, 30 mg/kg, i.p., 5 consecutive days). (F) Representative immunoblots of TH levels in the SN and STR after shAIMP1 stereotaxic injection. (G) Quantification analysis of TH expression levels in the SN shown in (F). (H) Quantification analysis of TH expression levels in the STR shown in (F). Unpaired *t* test. Data were expressed as mean ± SEM (*n* = 3–5). *p < 0.05, **p < 0.01, ***p < 0.001. Scale bar = 50 μm.

To study the functional involvement of AIMP1 in PD pathogenesis, stereotaxic delivery of lentivirus‐mediated shRNA targeting AIMP1 was performed in the SN, followed by systemic MPTP administration. Western blot was employed to assess the expression of AIMP1 and the results confirmed an approximately 50% reduction in AIMP1 protein levels compared to shRNA‐NC controls (Figure 2A–C). Immunostaining results indicated that AIMP1 knockdown did not significantly alter the number of dopaminergic (DA) neurons (Figure 2D). Behavioral assessment using the pole test revealed significant improvement in motor ability in AIMP1‐knockdown mice, as indicated by reduced latency to descend compared to MPTP‐treated controls (Figure 2E). Notably, AIMP1 knockdown led to a significant enhancement in the expression of TH in both the SN and striatum of PD mice (Figure 2F–H). These data indicated that AIMP1 knockout exerted neuroprotective effects in PD.

### 3.4 AIMP1 Modulated the Expression of Inflammation‐Associated Genes

**FIGURE 3:**
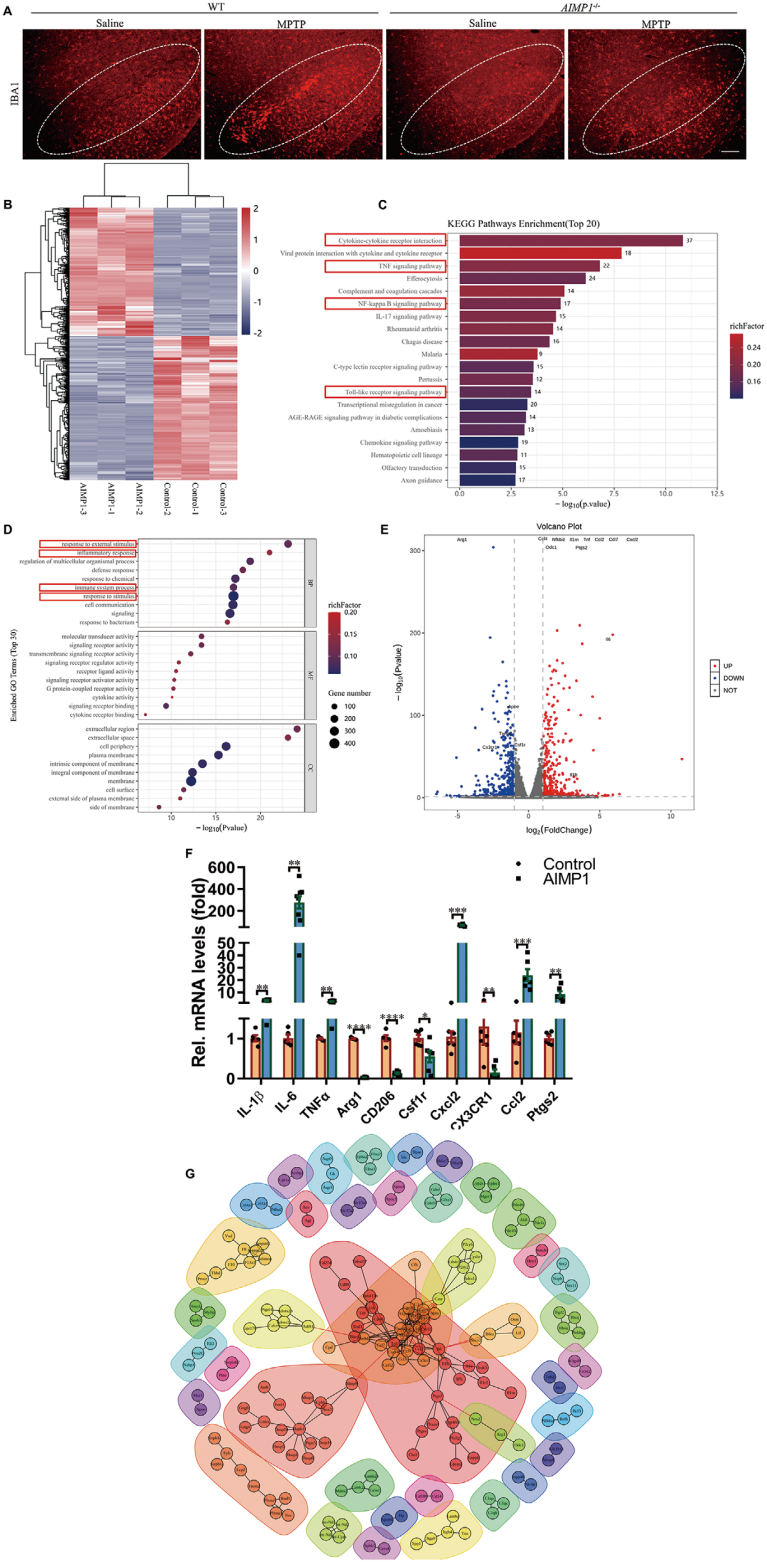
AIMP1 modulated neuroinflammation associated genes and pathways in microglia. (A) Representative images of IBA1 staining on SN in WT and *AIMP1* ^−/−^ mouse model of PD (MPTP, 20 mg/kg for male mice, 15 mg/kg for female mice, i.p., 4 times in 1 day). (B) Cluster analysis of the expression profile of the differentiated genes with Padjust < 0.05 & |log2FC| ≥ 1 in BV2 cells 24 h after AIMP1 treatment (UP: 420; Down: 471). (C) KEGG pathways enrichment analysis of the top 20 signaling pathways in BV2 cells induced by AIMP1. (D) GO Enriched analysis of differentiated gene expression in BV2 cells after AIMP1 treatment. (E) Volcano plot of RNA sequencing results in BV2 cells after AIMP1 treatment. (F) Verification of expression changes of selected targets. (G) Protein–protein interaction network analysis of the top 300 differentiated genes in BV2 cells induced by AIMP1. Nonparametric analysis (Mann–Whitney test) for IL‐1β and CX3CR1 in Figure 3F. *t* test for remaining data (*n* = 5–8). Data were expressed as mean ± SEM. *p < 0.05, **p < 0.01, ***p < 0.001. Scale bar = 50 μm.

To examined the regulatory role of AIMP1 in microglial activation, we performed IBA1^+^ microglial morphological analysis in MPTP‐treated PD mice. Notably, we observed that microglia exhibited pronounced activation in PD mice, which was attenuated by AIMP1 deficiency, as demarcated by the white dashed line in Figure 3A. To elucidate the molecular mechanisms underlying how AIMP1 regulated the microglial inflammatory response, the microglial cell line, BV2 cells, was treated with AIMP1 for RNA sequencing. It was revealed that numerous genes exhibited differential expression in the AIMP1‐treated group compared to the control group, among which there were 420 genes upregulated and 471 genes downregulated (with Padjust < 0.05 & |log2FC| ≥ 1) (Figure 3B). The differentially expressed genes were listed in Table S2 and Table S3. Enriched analysis highlighted significant associations with neuroinflammatory pathways, such as response to external stimulus, inflammatory response, immune system process, and response to stimulus (Figure 3C–D). For example, the expression of Csf1r, the important regulator of inflammation [17], significantly decreased in AIMP‐treated BV2 cells, whereas ptgs2, which has been reported to exhibit bidirectional regulation effects of inflammation [18], robustly increased (Figure 3E). To further validate the role of AIMP1 in modulating microglial inflammation, qPCR was conducted to analyze transcriptional alterations of target genes in Figure 3E. The qPCR analysis demonstrated that AIMP1 significantly upregulated pro‐inflammatory cytokines, including IL‐1β, IL6, and TNFα, while downregulated anti‐inflammatory markers Arg1 and CD206 (Figure 3F) in BV2 cells, thereby validating the RNA‐seq profiling trends. Protein–protein interaction (PPI) module analysis also demonstrated that AIMP1 induced the alterations of inflammation‐related genes in BV2 cells. For instance, the genes presented in Figure 3G, which were located at the core of the module, encoded inflammatory cytokines, including pro‐inflammatory factors like interleukin‐1β (IL‐1β), IL‐6, TNFα, as well as anti‐inflammatory factors such as arginase 1 (Arg1) and CX3CR1 (Figure 3G). These data collectively demonstrated that AIMP1 plays a vital role in regulating microglial‐associated inflammation accompanied by modulating inflammation gene expression.

### 3.5 AIMP1 Promoted the Expression of Microglial Inflammatory Cytokines

**FIGURE 4:**
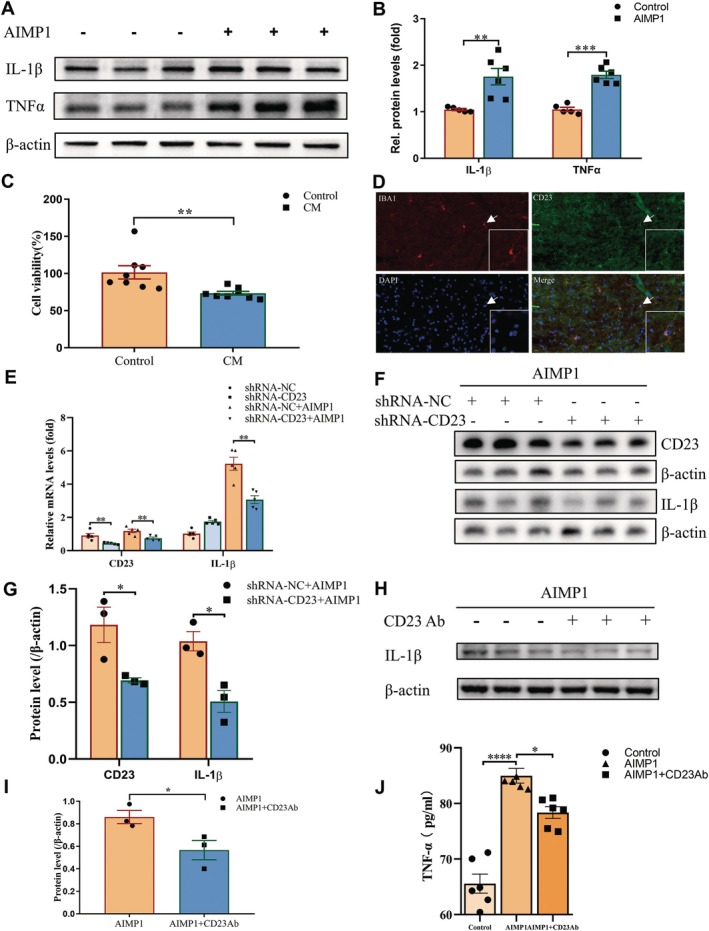
AIMP1 promoted microglial inflammatory response via CD23. (A) Representative blots of IL‐1β and TNFα of AIMP1‐treated BV2 cells (AIMP1: 0.5 μg/mL, 24 h). (B) Quantification analysis of IL‐1β and TNFα levels in (A). (C) MTT assay of SH‐SY5Y cells after treatment with CM from AIMP1‐treated BV2 cell (AIMP1: 0.5 μg/mL, 24 h). (D) Representative image of CD23 expression in microglia in midbrain of mice (adult WT mice following saline injection). The enlarged view of the indicated microglial cell pointed by white arrow was shown in the lower right corner. (E) qPCR analysis of IL‐1β and CD23 mRNA levels in shRNA‐CD23 or shRNA‐NC transfected BV2 microglial cells after AIMP1 treatment. (F) Representative blot of CD23 and IL‐1β protein expression levels in shRNA‐CD23 or shRNA‐NC transfected BV2 microglial cells after AIMP1 treatment. (G) Quantification analysis of CD23 and IL‐1β levels in (F). (H) Representative blots of IL‐1β in BV2 cells treated with AIMP1 alone or AIMP1 and CD23 antibody (AIMP1: 0.5 μg/mL, CD23 antibody: 0.5 μg, 24 h). (I) Quantification analysis of IL‐1β levels in (H). (J) Elisa assay revealed TNFα levels in supernatant of shRNA‐CD23 or shRNA‐NC transfected BV2 microglial cells after AIMP1 treatment. One‐way Anova analysis flowed by Tukey's multiple comparisons test for data in J, Unpaired *t* test for remaining data. Data were expressed as mean ± SEM (*n* = 3–8). *p < 0.05, **p < 0.01, ***p < 0.001. Scale bar = 20 μm.

To functionally validate the RNA sequencing findings on AIMP1‐mediated microglial inflammation, we also quantified inflammatory mediators in AIMP1‐stimulated BV2 cells by western blot. The results confirmed the increased protein levels of IL‐1β and TNFα (Figure 4A–B). These data indicated that AIMP1 induced a pro‐inflammatory microglial phenotype through bidirectional regulation of cytokine networks. Our findings support a pathogenic cascade wherein AIMP1 secreted by degenerating DA neurons activated microglia‐mediated neuroinflammation, subsequently exacerbating dopaminergic neurodegeneration in PD. Correspondingly, AIMP1 deficiency exhibited neuroprotective effects via suppressing microglia‐associated inflammation.

### 3.6 Conditioned Medium From AIMP1‐Treated BV2 Cells Induced the Death of SH‐SY5Y Cells

Next, we investigated whether AIMP1‐triggered microglia mediated neurotoxicity. The CM from AIMP1‐treated BV2 cells was collected and applied to the culture medium of SH‐SY5Y cells. MTT assay was performed at 24 h post treatment, and the results revealed a significant reduction in SH‐SY5Y cell viability exposed to AIMP1‐CM versus vehicle‐CM controls (Figure 4C). These findings indicated a causal link between AIMP1‐induced microglial inflammatory activation and neuronal degeneration. Taken together, these data strongly suggested that a pathogenic loop wherein AIMP1 secreted by damaged DA neurons drove microglia‐mediated neuroinflammation, which subsequently exacerbated dopaminergic neuronal loss in PD.

### 3.7 AIMP1 Regulated Microglia Inflammatory Response via CD23

Subsequently, we investigated the molecular mechanisms of AIMP1 in regulating microglial inflammation. Previous studies have indicated that AIMP1 binds CD23, a low‐affinity receptor for IgE, to regulate the inflammatory response in peripheral immune cells [19]. CD23 has been reported to be expressed in microglia cells [20, 21, 22]. To further verify the expression of CD23 in microglia, we performed immunostaining analysis. The results demonstrated CD23 expression in microglia localized within the midbrain of mice (Figure 4D). Building on prior evidence of AIMP1‐CD23 binding in peripheral immunity and microglial CD23 expression, we hypothesized that AIMP1 promotes microglial inflammation through this receptor. To test this hypothesis, we reduced CD23 expression by transfecting BV2 cells with shRNA‐CD23 lentivirus, which resulted in efficient CD23 knockdown (Figure 4E–G). Notably, AIMP1‐induced IL‐1β upregulation was abolished upon CD23 silencing (Figure 4E–G). Interestingly, CD23 antibody significantly attenuated the production of IL‐1β in AIMP1‐treated BV2 cells (Figure 4H–I) and reduced TNFα levels in the supernatant of AIMP1‐treated BV2 cells (Figure 4J). These data mechanistically established CD23 as a critical mediator of AIMP1‐triggered microglial activation, ultimately contributing to dopaminergic neuronal degeneration.

### 3.8 Specific Knockout of AIMP1 in Microglia or Astrocytes Had no Significant Effects on DA Neurons in PD

**FIGURE 5:**
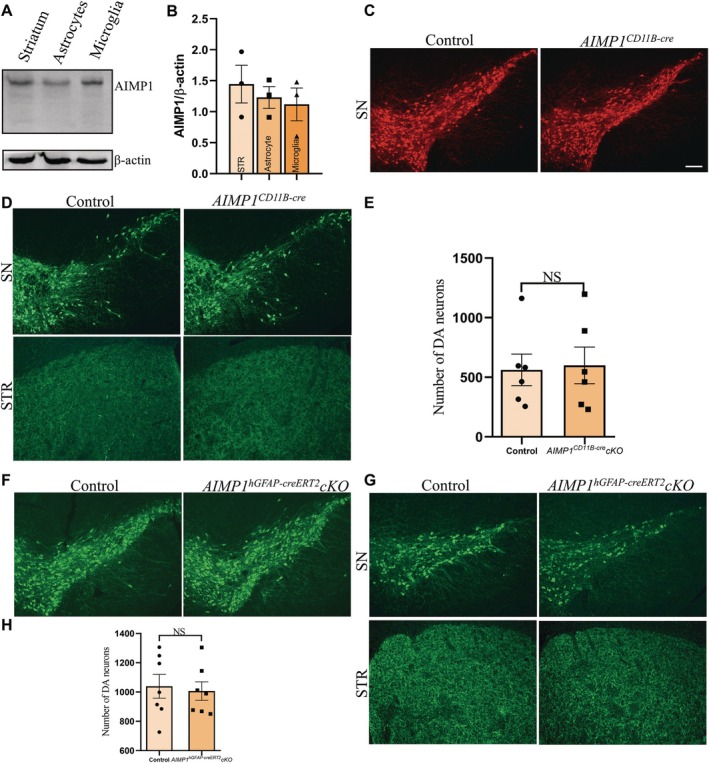
AIMP1 knockout in astrocytes or microglia had no effects on DA neurons. (A) AIMP1 was expressed in primary cultured microglia and astrocytes. (B) Statistical analysis of AIMP1 expression in (A). (C) Representative images of TH staining (red & green) on SN of adult control mice and AIMP1^CD11B‐cre^ mice. (D) Representative images of TH staining on SN and STR in MPTP‐induced PD mice after AIMP1 knockout in microglia (MPTP, 20 mg/kg for male mice, 15 mg/kg for female mice, i.p., 4 times 1 day). (E) Quantification analysis of DA neurons (TH^+^) number in the SN in (D). (F) Representative images of TH staining on SN of adult control mice and *AIMP1* ^*hGFAP‐creERT2*^ mice. (G) Representative images of TH staining on SN and STR in MPTP‐induced PD mice after AIMP1 knockout in astrocytes (MPTP, 20 mg/kg for male mice, 15 mg/kg for female mice, i.p., 4 times 1 day). (H) Statistical analysis of DA neurons in the SN in (G). Unpaired *t* test. Data were expressed as mean ± SEM (*n* = 3–7). *p < 0.05, **p < 0.01, ***p < 0.001. Scale bar = 50 μm.

Lastly, we aimed to elucidate whether AIMP1 derived from astrocytes or microglia was also responsible for the function of AIMP1 in PD. To address this question, we examined the expression of AIMP1 in astrocytes and microglia. The results showed that AIMP1 was expressed both in astrocytes and microglia (Figure 5A–B). Subsequently, we used *AIMP1* ^*CD11B‐cre*^ and *AIMP1* ^*hGFAP‐creERT2*^ conditional knockout mice to achieve selective AIMP1 ablation in microglia or astrocytes, respectively. We administered these mice with MPTP to establish a PD model and analyzed the impact on DA neurons. Interestingly, we observed that there were no significant differences in the number of DA neurons between *AIMP1* ^*CD11B‐cre*^ conditioned knockout mice and control mice following MPTP treatment (Figure 5C–E). Likewise, no significant alterations in the number of DA neurons were detected between *AIMP1* ^*hGFAP‐creERT2*^ conditioned knockout mice and control mice after MPTP treatment (Figure 5F–H). These results suggested that specific knockout of AIMP1 in microglia or astrocytes did not affect the number of DA neurons in PD mice, indicating that the effects associated with AIMP1 deficiency did not originate from astrocytes or microglia.

## 4 Discussion

**FIGURE 6:**
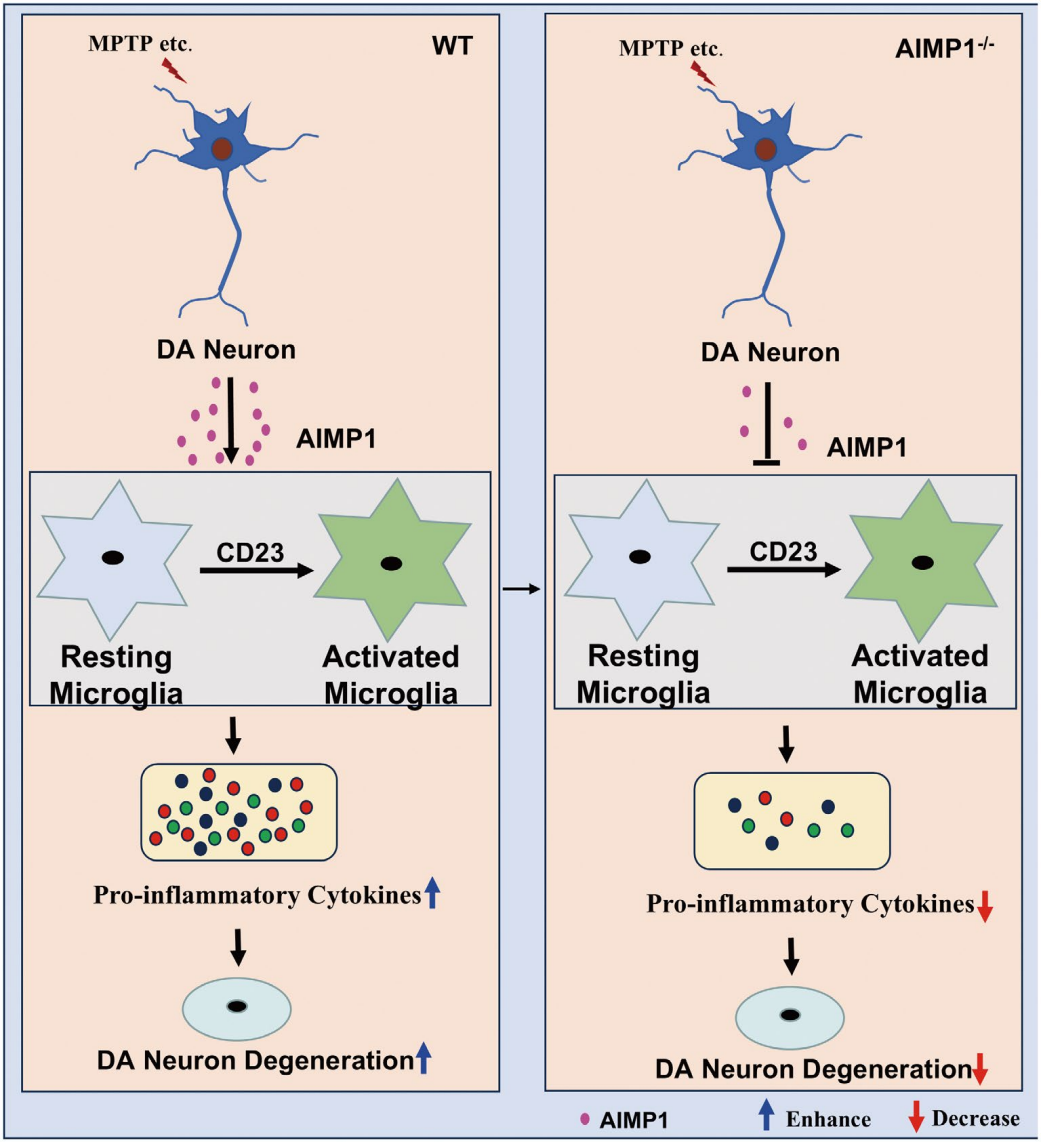
Schematic diagram of potential roles of AIMP1 in PD. Following MPTP treatment, AIMP1 could be secreted from DA neurons and induced the microglial activation accompanied by enhanced expression of pro‐inflammatory cytokines, leading to the neurodegeneration of DA neurons. AIMP1 knockout decreased the secretion of DA neurons derived AIMP1 and inhibited the CD23 dependent microglial inflammatory response, leading to neuroprotective effects in PD.

AIMP1, as an auxiliary factor of the large macromolecular aminoacyl tRNA synthetase complex, has been implicated in diverse pathologies including AD, systemic lupus erythematosus (SLE) and cancer [13, 23, 24, 25]. However, its pathophysiological role in PD remained undefined. In the present study, we revealed several key findings. First, we observed a significant elevation of AIMP1 in the blood of PD patients and the supernatant of the SH‐SY5Y cell line in an in vitro model of PD. Second, AIMP1 defects robustly enhanced behavioral and pathological performance in PD mice. Third, transcriptomic profiling analysis and cell experiments indicated that AIMP1 modulated the microglial inflammatory response in a CD23‐dependent manner. More intriguingly, specific knockout of AIMP1 in astrocytes or microglia had no significant effects on DA neurons in the PD mice model. Collectively, our data demonstrated that DA neuron‐derived AIMP1 drove microglia‐mediated neuroinflammation in PD (Figure 6). Thus, our studies provided novel insights into the underlying mechanisms of PD progression and potentially paved the way for future therapeutic interventions.

Previous research has demonstrated that AIMP1 was involved in neurodegenerative disease, including AD. Compared to the control group, levels of AIMP1 tend to be elevated in AD patients. Moreover, a significant correlation has been indicated between blood AIMP1 levels and the degree of medial temporal lobe atrophy (MTA) [13]. Additionally, studies have shown that suppressing AIMP1 could protect cognitive function in AD mice [13]. However, the role of AIMP1 in the pathogenesis of PD has not been fully elucidated. In the present study, we revealed a significant upregulation of AIMP1 levels in the blood of PD patients, consistent with previous observations in other neurodegenerative diseases. Behavioral tests indicated that AIMP1 inhibition improved motor ability in PD mice and increased the expression of TH in the SN, suggesting a neuroprotective role in PD. Furthermore, we demonstrated that global AIMP1 deficiency, rather than conditional knockout in astrocytes or microglia, protected DA neurons in the SN of the PD model. Collectively, these findings provided mechanistic evidence that neuron‐derived AIMP1 played a significant contributory role in PD pathology.

Previous investigations have established AIMP1 as a secretory product of peripheral immune cells to regulate the activation of peripheral cells [26]. In our current study, we discovered that MPP^+^ treatment significantly enhanced the AIMP1 levels in the culture medium of SH‐SY5Y cells. These findings indicated that AIMP1 could be secreted from neurons in the context of PD, leading to a significant elevation of blood levels of AIMP1. In other words, AIMP1 might serve as a crucial factor that bridges neurons and glial cells in PD pathology. It has been reported that neuron–microglia signaling is essential for brain function. For instance, neurons predominantly express certain ligands like CX3CL1 and CD200, which bind the corresponding receptors such as CX3CR1 and CD200R mainly expressed on microglia, thereby contributing to the regulation of brain immune homeostasis [27]. Moreover, the communication among neuronal cells plays important roles in elucidating the molecular mechanisms of PD. For example, our previous studies demonstrated that specific deletion of NG2 glia exacerbated microglial neuroinflammation and DA neuron loss in a PD mice model. Additionally, transforming growth factor‐β2 (TGFβ2) derived from NG2 glia regulated CX3CR1‐modulated immune response via its receptor TGFBR2 in microglia, highlighting the significant roles of the communication between NG2 glia and microglia in maintaining brain immune homeostasis in PD [28]. In this study, we showed that AIMP1 derived from DA neurons modulated neuroinflammation through CD23 in microglia, indicating that the AIMP1‐CD23 signaling pathway represents a novel connection between DA neurons and microglia in PD. Therefore, our study offers new perspectives on the mechanisms and treatment of PD from the viewpoint of neuron–glial communication.

Neuroinflammation was a crucial factor in the pathogenesis of PD [29]. Microglia were commonly considered the first line of defense against external stimuli and under pathological conditions [8, 30]. Inhibiting microglia‐associated neuroinflammation has been proposed as a potential therapeutic target for treating neuroinflammatory‐related brain disorders such as AD and PD [31, 32]. In our study, RNA sequencing demonstrated that AIMP1 significantly increased the expression of pro‐inflammatory factors such as IL‐1β and IL‐6, while downregulating the expression of anti‐inflammatory factors such as Arg1. These findings were further validated by quantitative qPCR and western blot analyses. Prior studies have demonstrated CD23 expressed in microglia [20, 21, 22]. Notably, elevated CD23 levels were observed in PD patient‐derived microglia, suggesting its functional involvement in PD‐associated neuroinflammatory pathways. In this study, multiple methods were employed to confirm that CD23 suppression significantly attenuated microglia‐associated neuroinflammation. Collectively, our results indicated that AIMP1‐CD23 signaling was a key factor in promoting microglia activation. Consequently, inhibiting the AIMP1‐CD23 pathway could effectively alleviate pro‐inflammatory reactions and enhance anti‐inflammatory responses, offering valuable insights for the development of anti‐inflammatory drugs for PD treatment.

In summary, the current study demonstrated that AIMP1, secreted by DA neurons, modulated the CD23‐dependent microglial inflammatory response, thereby contributing to the progression of PD. Thus, this research not only underscored the significant role of AIMP1 in neurodegenerative disorders, but also proposed that blockade of AIMP1‐CD23 signaling might represent a viable therapeutic approach for the treatment of PD.

## Author Contributions

Q.W. and X.Y. conducted most of the experiments, X.M. and R.L. contributed to the clinical experiments. H.Y. contributed to the manuscript writing and data analysis. X.L. and X.W. contributed to the cell culture. S.Y. contributed to the editing of the manuscript. All the authors approved the final manuscript.

## Conflicts of Interest

The authors declare no conflicts of interest.

## Supporting information


**Table S1.** General information of human participants.
**Table S2.** Upregulated genes identified by RNA‐Seq analysis.
**Table S3.** Downregulated genes identified by RNA‐Seq analysis.

Data S1.

Data S2.

Figure S1.

## Data Availability

The data that support the findings of this study are available from the corresponding author upon reasonable request.
