# Supplementary figures and images for: Dopaminergic Neuron‐Derived AIMP1 Promotes Neurodegeneration via CD23‐Dependent Microglial Activation
Source: CNS Neurosci Ther. 2025 Jun 16;31(6):e70472. doi: 10.1111/cns.70472 (PMC12168485; doi:10.1111/cns.70472)

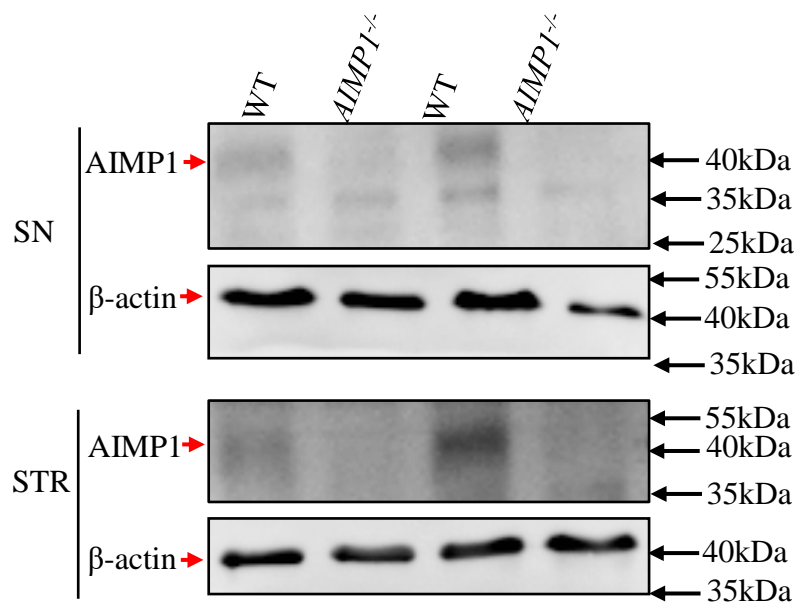

**Figure 1D**

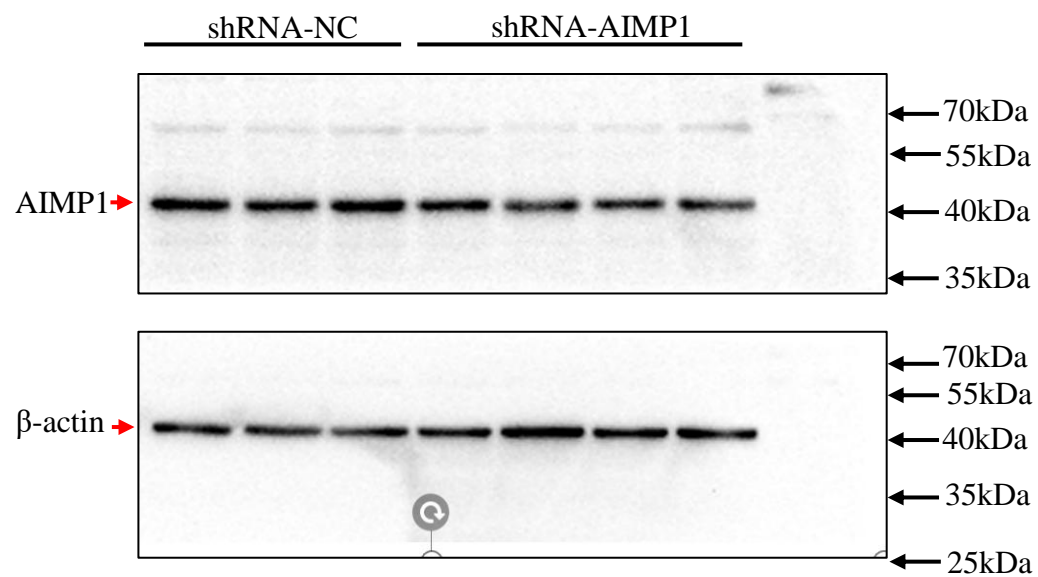

**Figure 2B**

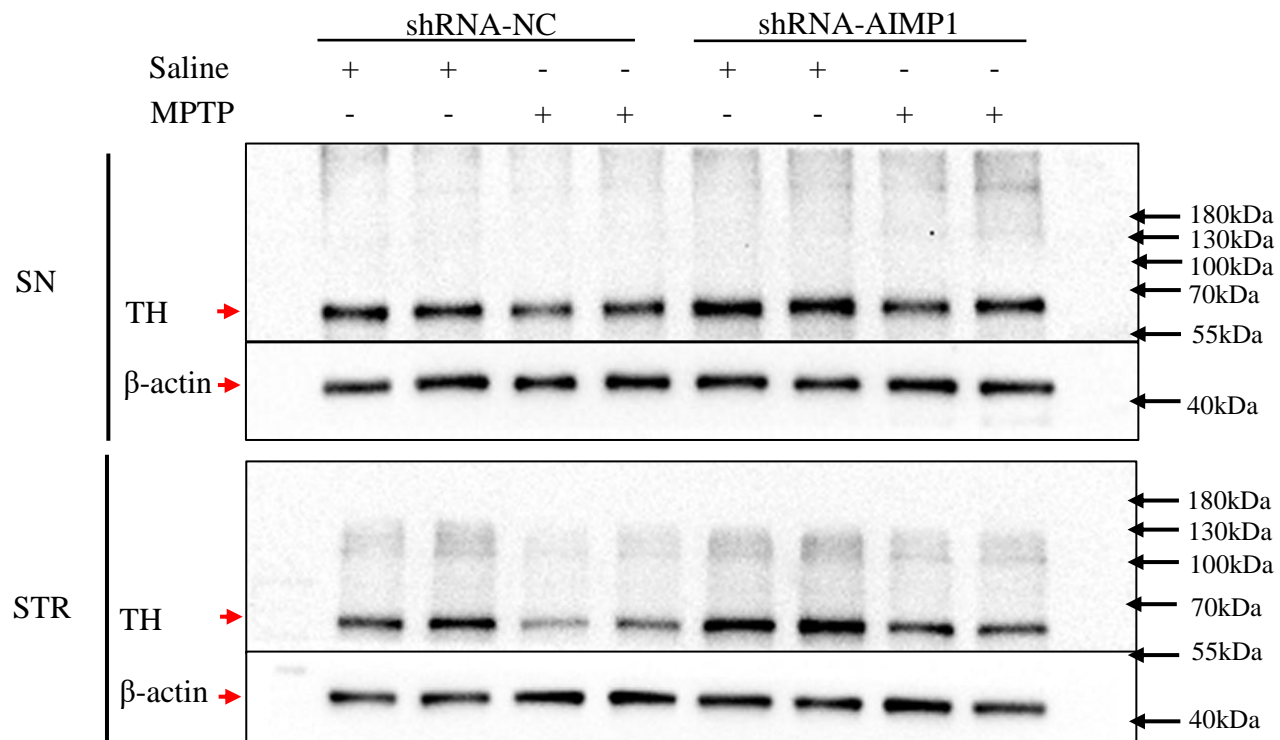

**Figure 2F**

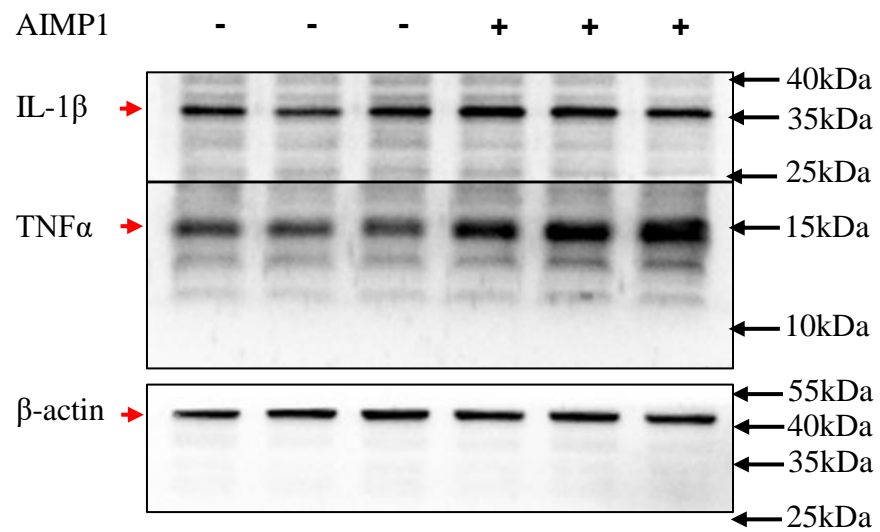

**Figure 4B**

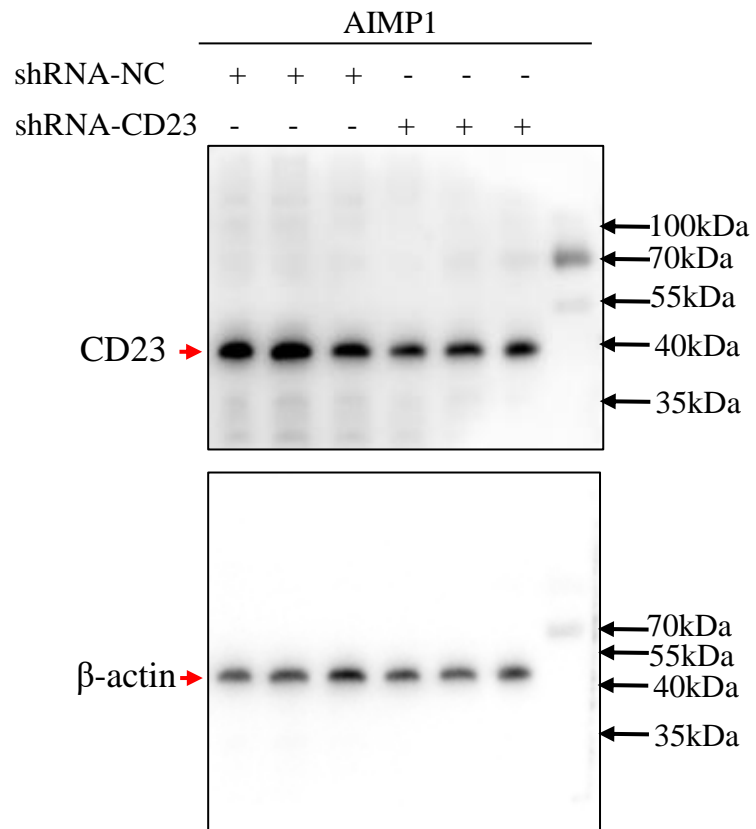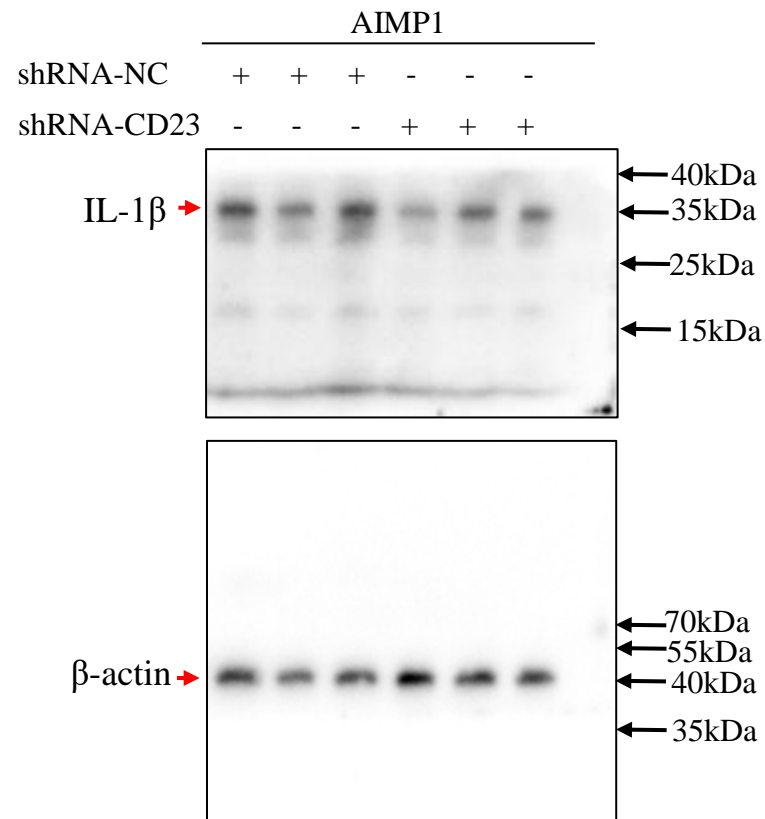

**Figure 4G**

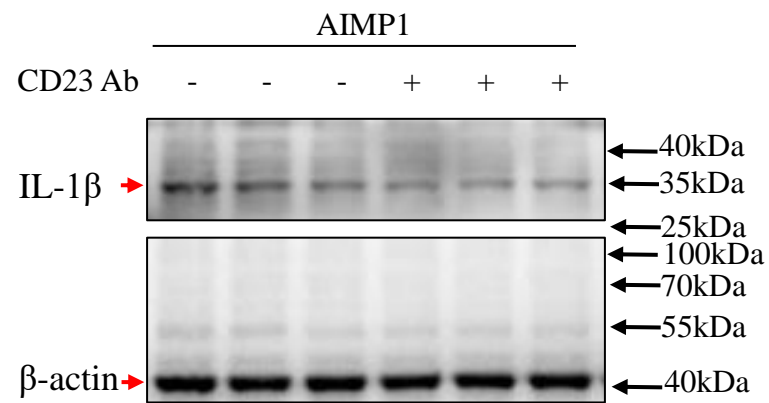

**Figure 4I**

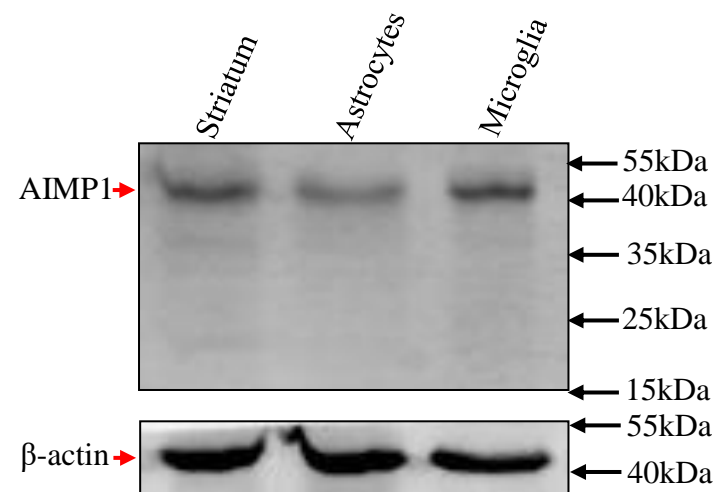

**Figure 5A**

Supplement: Supplementary file 4 — Figure S1. [file CNS-31-e70472-s003.pdf]
